# Prognostic nutritional index and naples prognostic score as biomarkers for the prognosis of incisional wound healing after thoracolumbar tuberculosis surgery

**DOI:** 10.1371/journal.pone.0309267

**Published:** 2024-12-13

**Authors:** Tuotuo Xiong, Wanyuan Qin, Ye Zhang, Yuxing Chen, Yunsheng Ou

**Affiliations:** 1 Department of Orthopaedic Surgery, The First Affiliated Hospital of Chongqing Medical University, Chongqing, China; 2 Chongqing Municipal Health Commission Key Laboratory of Musculoskeletal Regeneration and Translational Medicine, Chongqing, China; 3 Orthopaedic Research Laboratory of Chongqing Medical University, Chongqing, China; 4 Department of Orthopaedic Surgery, Chongqing university Jiangjin hospital, Chongqing, China; Shuguang Hospital, CHINA

## Abstract

**Objectives:**

This study aimed to evaluate and compare the clinical predictive value of prognostic nutritional index (PNI) and naples prognostic score (NPS) as biomarkers for the prognosis of incisional wound healing in patients who underwent thoracolumbar tuberculosis surgery through the posterior approach.

**Methods:**

From January 2019 to October 2021, a total of 124 patients with thoracolumbar tuberculosis who underwent posterior approach debridement and internal fixation were included in this study. We retrospectively analyzed the clinical data, including PNI and NPS. They were divided into poor wound healing (PWH) and non-PWH groups according to whether PWH developed after the operation. And according to the receiver operating characteristic curve, patients were divided into two groups through the threshold value. Risk factors were found using logistic regression analysis.

**Results:**

The unfavorable outcome group had lower hemoglobin, serum albumin, Pre-albumin, PNI, and higher estimated blood loss, instrumented segments, neutrophil count, and NPS (P < 0.05). Both PNI and NPS were strongly correlated with PWH (r = 0.373, P < 0.05; r = −0.306, P < 0.05, respectively). The area under the curve (AUC) of PNI for predicting unfavorable outcomes was 0.764 (95% CI 0.662–0.865, P < 0.001), which was similar to NPS (0.808, 95% CI: 0.719–0.897, P < 0.001). Multivariate stepwise logistic regression analysis showed that PNI, NPS, the neutrophil count, the level of serum albumin, and the number of instrumented segments were independent risk factors for PWH.

**Conclusion:**

Both PNI and NPS might be novel independent biomarkers and predictors of poor outcomes in incisional wound healing after STB surgery.

## Introduction

Tuberculosis (TB) is one of the oldest diseases known to people, and this is evident from the characteristic lesions on the bones of Neolithic humans, which have plagued humans since prehistoric times, also known as Porter’s disease [1, 2]. An estimated 2 billion people worldwide are infected with TB, extrapulmonary TB is found in 20% of infected people, and skeletal muscle TB is seen in nearly 10% of dynamic TB patients. The spine is the most common site, which accounts for 50% of patients with bone tuberculosis [3–6]. The onset of spinal tuberculosis is slow and insidious, and patients present with or without typical back pain symptoms. If not diagnosed and treated promptly, kyphosis and serious neurological complications may occur. Paraplegia, the most dreaded complication, occurs in 10 to 30 percent of such patients [5, 7–9]. The thoracic vertebrae (40%-50%) and lumbar vertebrae (35%-45%) are common involved parts of the spine [10]. In clinical practice, spinal TB patients with clear surgical indications should first receive standard anti-tuberculosis drug treatment, followed by surgical treatment, including abscess drainage, lesion removal, bone graft fusion, and internal fixation, so as to improve the efficacy of anti-tuberculosis drugs, enhance tuberculosis control, promote bone graft fusion, and rebuild the stability of the spine [11–13]. TB attacks vulnerable populations, targeting patients with immune dysfunction, malnutrition, diabetes, smoking, alcohol, etc [14, 15]. As a result, they are prone to different postoperative complications than patients with spinal degenerative diseases [16, 17]. Poor wound healing (PWH) is a fatal surgical complication and a major cause of postoperative surgical site infection (SSIs), which can lead to serious outcomes such as failure of internal fixation, pseudarthrosis, osteomyelitis, prolonged hospital stay, increased mortality, and economic costs, and even secondary revision surgery [18–20]. Tuberculosis control and nutritional status are two key factors in the treatment of spinal tuberculosis, and nutritional status also affects the healing of postoperative incisions [21]. Therefore, patients should be evaluated for nutrition at admission using relevant markers to predict their potential for poor postoperative incision outcomes. Various assessment methods have been recommended, such as the NRS-2002 score and the Patient-Generated Subjective Global Assessment (PG-SGA). Still, these methods are limited because they include multiple subjective factors and require expert knowledge to test accurately. The Prognostic nutritional index (PNI), based on serum albumin and peripheral blood lymphocyte counts, was originally used to assess preoperative nutritional status, surgical risk, and postoperative complications in patients undergoing surgery. It has been shown to be a prognostic biomarker for postoperative spinal infection, postoperative delirium, solid tumors, and cardiovascular disease [22]. The Naples prognostic score (NPS), first described by Gennaro et al [23] as an independent prognostic factor in patients with colorectal cancer surgery, contains four nutritional and immune markers: serum albumin level, total cholesterol level, NLR, and LMR, and has been proved a good prognostic effect in gastrointestinal tumors [24]. However, the role of PNI and NPS in incision prognosis after spinal thoracolumbar tuberculosis is unclear. Therefore, the purpose of this study was to investigate and compare the relationship between nutritional status assessed by PNI and NPS and postoperative incisional wound prognosis.

## Methods

All participants signed a written informed consent to participate in this study before data was stored in a hospital database and used for study purposes and underwent standardized anti-tuberculosis therapy before surgery.

### Patient selection

A retrospective analysis was performed on 124 patients with STB in our hospital from January 2019 to October 2021 who underwent a posterior approach of thoracolumbar tuberculosis debridement, bone graft fusion, and internal fixation. It’s all done with the same surgeon and the same technique. We accessed the data for research purposes on September 1^st^, 2022. We had access to information that could identify individual participants during or after data collection.

#### Inclusion criteria

(a) Complete medical records, including clinical data on general conditions, perioperative laboratory tests, imaging findings, and perioperative clinical features; (b) Patients undergoing STB surgery for the first time; (c) Postoperative pathological diagnosis of the patient was STB; (d) No diseases affecting the blood test.

#### Exclusion criteria

(a) suspected STB but not confirmed by pathological examination, (b) preliminary and pathological diagnosis of non-STB, (c) previous STB surgery history, (d) younger than 18 years of age, (e) serious heart, liver, kidney insufficiency, (f) blood diseases and (g) incomplete clinical data.

### Perioperative management

#### Preoperative management

According to our previous clinical experience and research, all patients were treated with oral administration of standardized the HREZ chemotherapy regimen daily for 2–4 weeks before surgery. Diabetes mellitus, coronary heart disease, hypertension and other basic underlying diseases were consulted by the appropriate corresponding departments before the operation, and individualized diagnosis and treatment was carried out to adjust the blood glucose, blood pressure and blood lipids to operable standards. All patients were scheduled for surgical treatment after dynamic monitoring of no obvious abnormalities. [25]

#### Surgical procedure

After endotracheal intubation and general anesthesia, the patient was in a prone position with the abdomen suspended, and the C-arm was used to identify the diseased segment. The posterior median incision was made with the lesion segment as the center, and the bilateral paravertebral muscle was detached through the posterior median subperiosteal approach (if the lesion was biased to one side, the contralateral intermuscular approach was performed). The spinous process, lamina, the upper and lower articular process of the diseased vertebra, and adjacent normal vertebra were revealed. Pedicle screws were implanted into one or two vertebrae adjacent to the affected vertebrae, and pedicle screws were implanted on both sides of the affected segment (only the opposite side of the pedicle screw was inserted when one vertebra was severely invaded), and titanium rods were temporarily locked for temporary fixation. According to the degree of lesion destruction, a transforaminal or pedicle approach was performed to remove the anterior column and surrounding tuberculosis lesions from the lateral and posterior, including pus, dead bone, residual intervertebral disc tissue, and caseous necrotic tissue. Relatively healthy bone tissue was retained until the bone surface hemorrhage and intraspinal decompression was performed. Autogenous granular bone mixed with isoniazid 0.3g and streptomycin 1g were implanted, iliac bone pieces or titanium cages filled with autogenous bone, and the posterior margin of the vertebral body was covered with a gelatin sponge containing isoniazid to prevent the entry of bone graft particles into the spinal canal. The kyphotic deformity was corrected with the fixed screw after proper pressure. The C-arm X-ray machine showed deformity correction. After the position of the inner plant was satisfied, the connecting rod was finally fixed. Two drainage tubes were placed in the incision, and the incision was closed in layers.

#### Routine postoperative management

All patients were treated with cefuroxime sodium 1.5g bid for prophylactic anti-infection three days after surgery, and the drainage tube was removed when the daily drainage volume was less than 40ml/d, and an X-ray examination was performed after extubation. Standardized anti-tuberculosis therapy was continued for 18 to 24 months after surgery. The patient can get out of bed with the protective gear one week after surgery.

### Measures and statistics

#### Data collection

Within one month after surgery, the wound healing process is obviously stagnant or delayed, leading to a long-term non-healing state, which may be complicated by exudate, wound hematoma, and even incision dehiscence, which can be defined as PWH. Based on previous studies and our experience, we evaluated the following items that might be used to analyze postoperative PWH in patients with STB, Preoperative basic clinical information of patients, preoperative laboratory indicators, and operation-related objects. Basic clinical characteristics include Sex, age, BMI, smoking history, drinking history, and whether there is a combination of hypertension, pulmonary tuberculosis, or diabetes. Laboratory measures include neutrophil count, lymphocyte count, monocyte count, total cholesterol, hemoglobin, prealbumin, and albumin, laboratory tests were performed 1 to 2 days before surgery. The surgery-related parameters included the number of fixed segments and intraoperative estimated blood loss. PNI was calculated using the following formula: 10 × serum albumin (g/dL) + 0.005 × total lymphocyte count (/mm^3^). NPS is based on the following four parameters: serum albumin (g/dL), total cholesterol (mg/dL), lymphocyte-monocyte ratio (LMR), and neutrophil-lymphocyte ratio (NLR). (Table 1).

**Table 1:** Evaluation of the nutrition status utilizing the NPS.

Alb(g/L)	score	TG(mg/dL)	score	NLR	score	LMR	score
≥40	0	>180	0	≤2.96	0	>4.44	0
<40	1	≤180	1	>2.96	1	≤4.44	1
Total Score: 0-None; 0~2-Light; >2-Severe							

#### Statistical analysis

Continuous data are expressed as mean ± standard deviation (SD) or median (quartile range), and categorical data are expressed as frequency and percentage (%). Chi-square test, independent sample T test, or non-parametric test of two independent samples were selected to carry out a single factor analysis of independent variables, respectively, comparing categorical variables and continuous variables. After the Shapiro-Wilk normality test, continuous variables conform to the normal distribution of selected independent samples T-test, and non-normal distribution variables are assigned two independent samples non-parametric test. The receiver operating characteristic curve (ROC) analysis determined the threshold of the continuous variable. We used the area under the curve (AUC) to assess the predictive value, comparing AUC using the DeLong method. The prevalence of included clinical features was assessed by calculating the sensitivity and specificity of each factor. Correlation analysis of data with normal distribution used Pearson test, while Spearman test was used for data with nonnormal distribution. Univariate analysis and multivariate logistic regression were used to determine independent predictors of PWH. P <0.05 was considered statistically significant. SPSS 26.0 statistical software and MedCalc statistical software 19.0.7 version were used for statistical analysis.

### Ethical approval

The authors are accountable for all aspects of the work in ensuring that questions related to the accuracy or integrity of any part of the work are appropriately investigated and resolved. This study was conducted in accordance with the Declaration of Helsinki and was approved by the Institutional Ethics Board of the First Affiliated Hospital of Chongqing Medical University.

## Results

### Baseline characteristics

**Fig 1:**
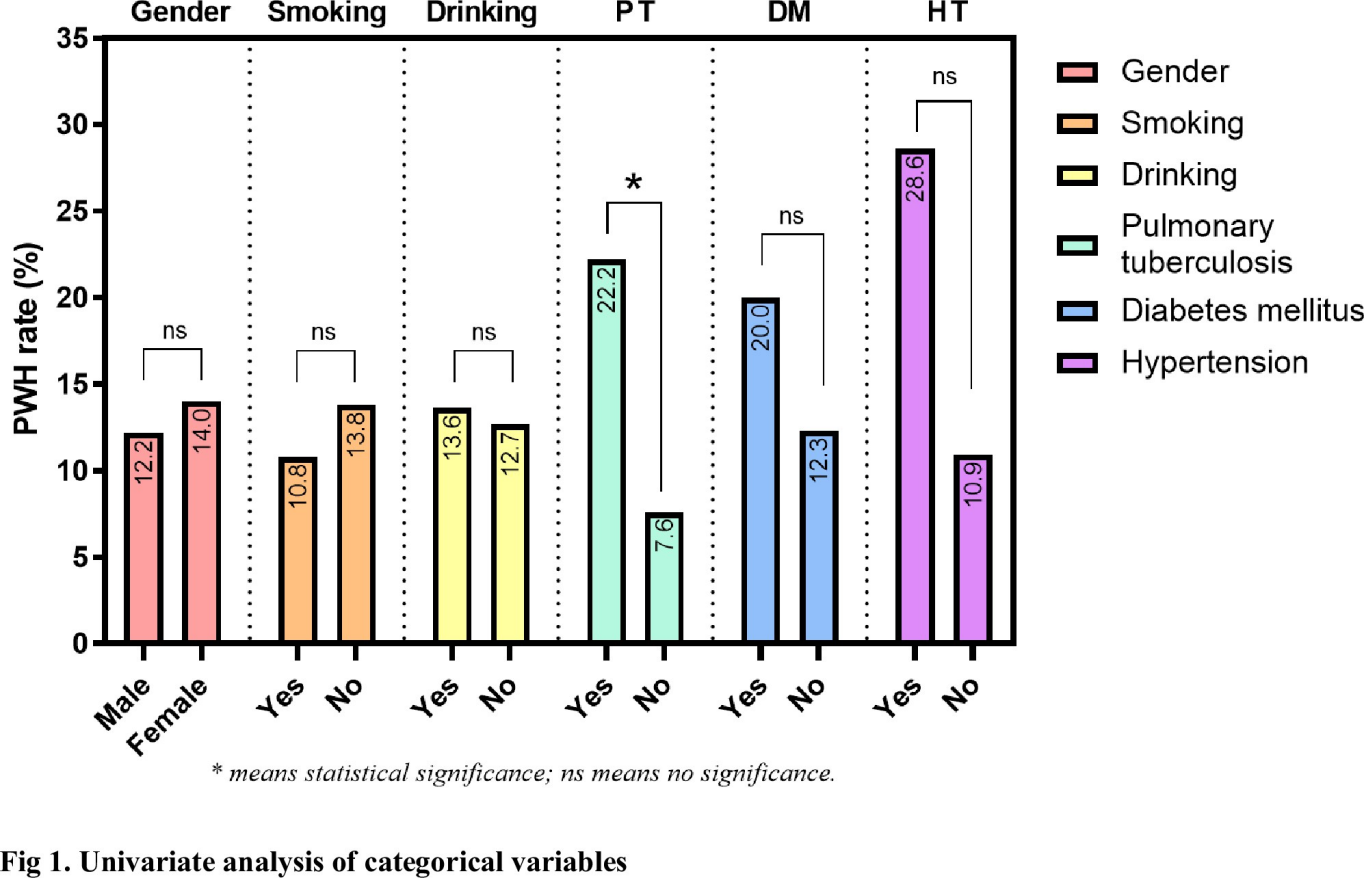
Univariate analysis of categorical variables.

**Fig 2:**
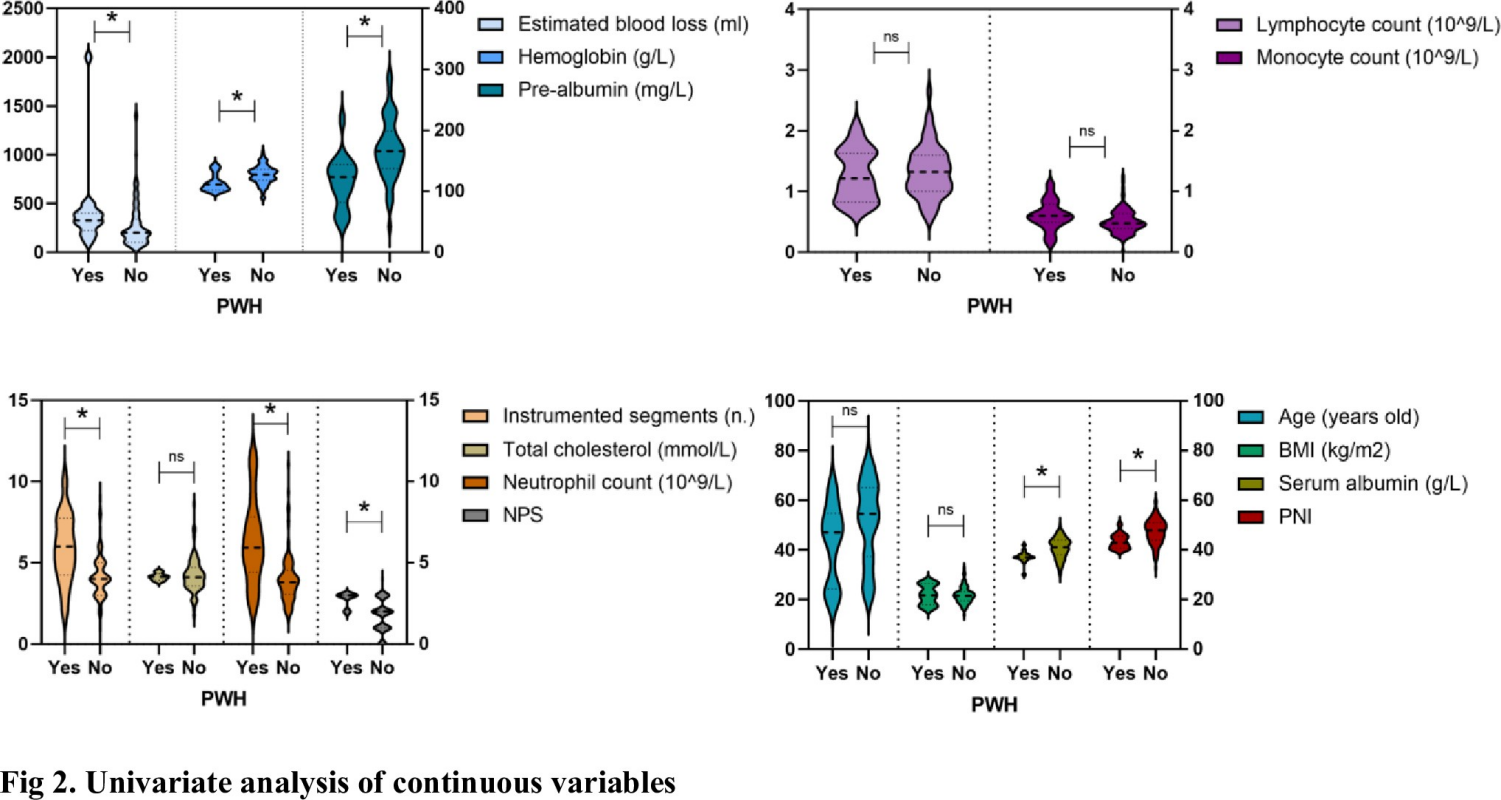
Univariate analysis of continuous variables.

**Fig 3:**
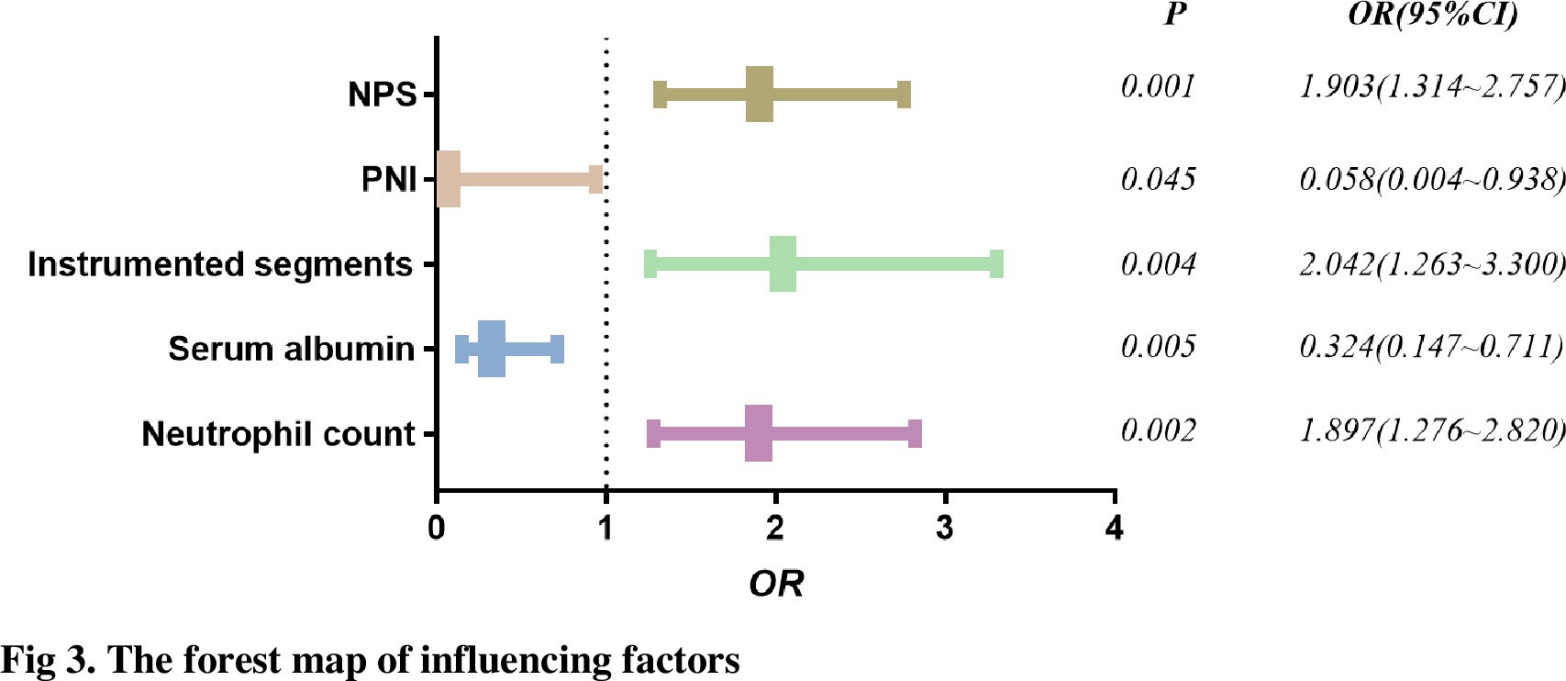
Receiver operating characteristic (ROC) analysis of PNI and NPS.

**Fig 4:**
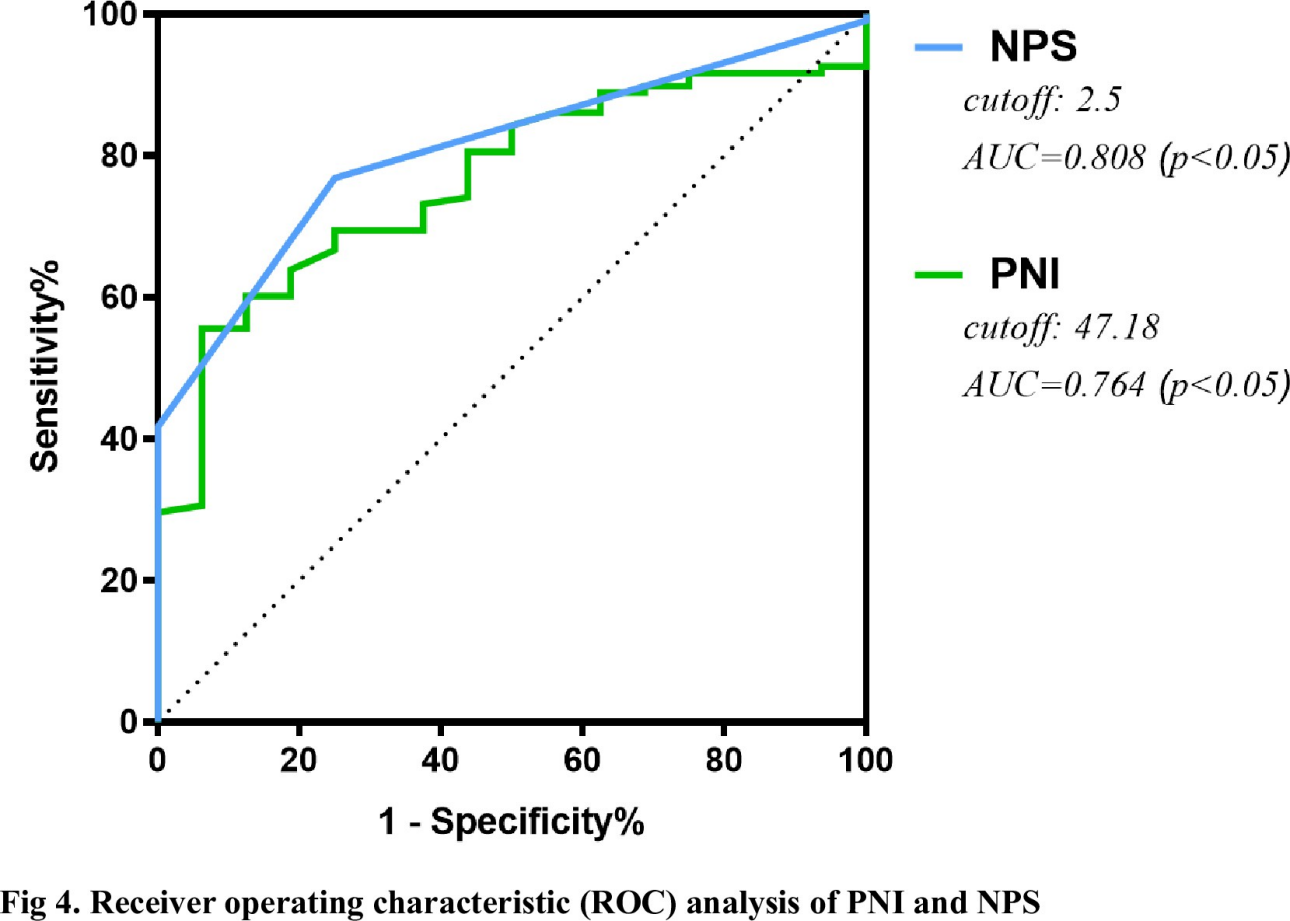
ROC analysis of significant independent factors other than PNI and NPS.

A total of 124 patients with thoracolumbar tuberculosis who underwent posterior approach thoracolumbar tuberculosis debridement and internal fixation were included in this study. The median age of the PWH group and non-PWH group was 54.5 years (37.25–65) and 49 years (39.75–57.25), respectively. The median BMI was 21.5(19.48–23.17) and 21.1(17.9–25.88), respectively. 108 patients (65 males and 43 females) did not develop PWH. Among the remaining 14 patients with PWH, 9 were males and 7 were females, 4 cases had a smoking history, 3 had a drinking history, 10 were complicated with pulmonary tuberculosis, 3 were with diabetes, and 4 were with hypertension. (Figs 1 and 2; Table 2) Through the receiver operating characteristic curve (ROC) analysis, the patients were divided into a high PNI group and a low PNI group according to the PNI cut-off value of 47.18 (Fig 3), 61 patients were divided into low PNI group, and the remaining 63 cases were included in high PNI group [26, 27]. The number of cases of pulmonary tuberculosis in the high PNI group was significantly higher than that in the low PNI group (16 cases and 29 cases), and the age in the high PNI group was significantly higher than that in the low PNI group (48 and 58 years). The included patients were also divided into the low NPS group and the high NPS group according to the NPS cut-off value of 2.5 (Fig 4), and their clinical characteristics were similar to those of the PNI group. (Tables 3 and 4).

**Table 2:** Univariate analysis of included patients by clinical characteristics.

Characteristic		PWH		*χ*^2^/ *Z*	*P*
		No	Yes		
Sex	Male	65	9	0.090	0.765
	Female	43	7		
Smoking	No	75	12	0.205	0.650
	Yes	33	4		
Drinking	No	89	13	0.013	0.910
	Yes	19	3		
Pulmonary tuberculosis	No	73	6	5.458	**0.019** *
	Yes	35	10		
Diabetes mellitus	No	99	13	0.487	0.485
	Yes	9	3		
Hypertension	No	98	12	3.447	0.063
	Yes	10	4		
Age (years old)		54.5(37.25~65)	49(39.75~57.25)	-0.984	0.325
BMI (kg/m^2^)		21.5(19.48~23.17)	21.1(17.9~25.88)	-0.205	0.838
Instrumented segments (n.)		4(3~5)	6(5~8)	-3.407	**0.001** *
Estimated blood loss (ml)		200(100~337.5)	325(225~400)	-2.404	**0.016** *
Neutrophil count (10^9/L)		3.8(3.03~4.6)	5.6(3.77~6.79)	-3.723	**<0.001** *
Lymphocyte count (10^9/L)		1.3(1~1.6)	1.2(0.83~1.63)	-0.548	0.584
Monocyte count (10^9/L)		0.5(0.39~0.63)	0.6(0.49~0.79)	-1.860	0.063
Total cholesterol (mmol/L)		4.12(3.58~4.73)	4.14(3.94~4.34)	-0.079	0.937
Hemoglobin (g/L)		127.5(118.75~136)	111(102.25~123.25)	-3.225	**0.001** *
Pre- albumin^#^ (mg/L)		169.65±51.18	115.94±43.22	3.988	**<0.001** *
Serum albumin (g/L)		41(38~44)	37(36.25~38)	-3.562	**<0.001** *
PNI^#^		47.46±5.01	43.45±2.99	-4.515	**<0.001** *
NPS		2(1~2)	3(2.25~3)	-4.135	**<0.001** *

^#^ indicates that the SW normality test follows a normal distribution.

* means statistical significance.

**Table 3:** Clinical characteristics of the low-PNI and high-PNI.

Characteristic		PWH		*χ* ^2^ */Z*	*P*
		Low-PNI	High-PNI		
Sex	Male	39	35	0.904	0.342
	Female	22	28		
Smoking	No	44	43	0.223	0.637
	Yes	17	20		
Drinking	No	49	53	0.307	0.580
	Yes	12	10		
Pulmonary tuberculosis	No	45	34	5.256	**0.022** *
	Yes	16	29		
Diabetes mellitus	No	55	59	0.508	0.476
	Yes	6	4		
Hypertension	No	54	58	0.444	0.505
	Yes	7	5		
Age (years old)		48(27.5~56.5)	58(48~66)	-3.84	**<0.001** *
BMI (kg/m^2^)		21.64(19.88~24.28)	21.41(18.82~23.24)	-1.095	0.274
Instrumented segments (n.)		4(3~5)	4(4~6)	-1.965	**0.049** *
Estimated blood loss (ml)		200(100~300)	300(150~400)	-2.31	**0.021** *
Neutrophil count (10^9/L)		3.7(2.99~4.25)	4.24(3.19~5.38)	-2.169	**0.030** *
Lymphocyte count (10^9/L)		1.46(1.2~1.69)	1.14(0.9~1.38)	-4.166	**<0.001** *
Monocyte count (10^9/L)		0.47(0.37~0.64)	0.49(0.42~0.63)	-1.217	0.223
Total cholesterol (mmol/L)		4.18(3.63~4.93)	4.12(3.54~4.41)	-.1.183	0.237
Hemoglobin (g/L)^#^		117.83±13.83	133.28±13.374	-6.322	**<0.001** *
Pre- albumin (mg/L)		180(153.5~228.5)	139(102~168)	-5.478	**<0.001** *
Serum albumin^#^ (g/L)		37.13±2.803	43.61±2.347	-13.935	**<0.001** *

^#^ indicates that the SW normality test follows a normal distribution.

* means statistical significance.

**Table 4:** Clinical characteristics of the low-NPS and high-NPS.

Characteristic		NPS		*χ* ^2^ */Z*	*P*
		Low-NPS	High-NPS		
Sex	Male	54	20	0.693	0.405
	Female	33	17		
Smoking	No	65	22	2.885	0.089
	Yes	22	15		
Drinking	No	71	31	0.084	0.772
	Yes	16	6		
Pulmonary tuberculosis	No	60	19	3.484	0.062
	Yes	27	18		
Diabetes mellitus	No	81	33	0.536	0.464
	Yes	6	4		
Hypertension	No	79	33	0.078	0.781
	Yes	8	4		
Age (years old)		52(35~63)	54(42~66.5)	-1.328	0.184
BMI (kg/m^2^)^△^		22.04±3.52	21.15±2.93	-1.35	0.179
Instrumented segments (n.)		4(3~5)	4(4~6)	-2.124	**0.034** *
Estimated blood loss (ml)		200(100~300)	300(200~450)	-2.588	**0.010** *
Neutrophil count (10^9/L)		3.7(2.91~4.24)	5.07(3.94~6.79)	-4.517	**<0.001** *
Lymphocyte count (10^9/L)		1.38(1.14~1.66)	1.01(0.78~1.35)	-3.668	**<0.001** *
Monocyte count (10^9/L)		0.47(0.38~0.63)	0.53(0.44~0.71)	-1.781	0.075
Total cholesterol (mmol/L)		4.21(3.65~4.79)	3.98(3.51~4.33)	-1.473	0.141
Hemoglobin^△^ (g/L)		129.02±14.8	116.97±14.32	-4.188	**<0.001** *
Pre- albumin (mg/L)		173(150~218)	121(83.5~147)	-6.335	**<0.001** *
Serum albumin (g/L)		43(40~44)	37(35~38)	-7.238	**<0.001** *

^#^ indicates that the SW normality test follows a normal distribution.

* means statistical significance.

### Association between PNI, NPS and PWH

Sixteen (12.9%) patients in our study had the PWH one month after surgery, and fifteen (93.8%) of them had a low PNI. Plus, twelve (87.5%) patients had a high NPS in the unfavorable outcome group. Spearman correlation analysis showed that both NPS and PNI were strongly correlated with the PWH after the procedure (r = 0.373, P < 0.05; r = −0.306, P < 0.05, respectively). Moreover, there was also a significant correlation between PNI and NPS according to Spearman correlation analysis (r = −0.783, P < 0.05). The high PNI group and low NPS group were highly overlapping (Table 5).

**Table 5:** Relationship between PNI and NPS score.

		NPS	
		Low	High
PNI	High	60	1
	Low	27	36

### Effect of PNI and NPS on outcome

**Fig 5:**
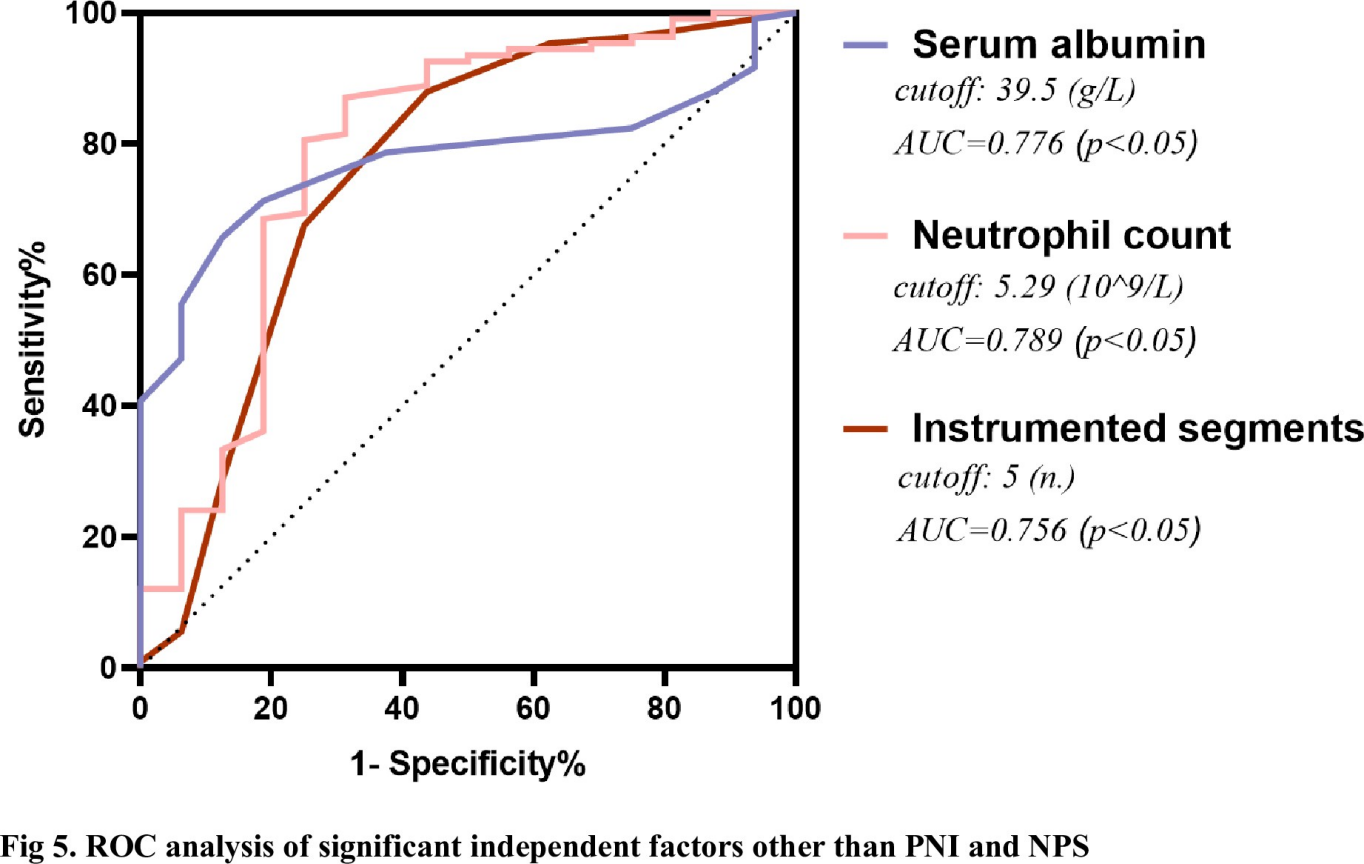
The forest map of influencing factors.

The receiver operating characteristic curve for unfavorable outcomes showed that the PNI and the NPS had predictive value. The PNI predicted an unfavorable prognosis with an AUC of 0.764 (95% CI 0.662–0.865, P < 0.001), which was similar to NPS (0.808, 95% CI: 0.719–0.897, P < 0.001) (Fig 3). The sensitivity and specificity of expected performance were 93.8% and 55.56% for the PNI and 75.0% and 76.85% for the NPS, respectively. The De Long method showed no significant difference in AUC between the CONUT and PNI (P = 0.329). As mentioned above, when compared with the favorable outcome group, patients with higher NPS and lower PNI (P < 0.001) were observed in the unfavorable outcome group. (Table 6) To further investigate the relationship between the PWH and the basic clinical characteristics, the results explored by a univariate analysis showed patients with preoperative pulmonary tuberculosis, higher neutrophil count, lower hemoglobin, lower prealbumin, lower albumin, more instrumented segments, and more intraoperative estimated blood loss were more likely to develop PWH within one month after surgery (P < 0.05). Furthermore, multivariate analysis showed that the PNI and the NPS were two independent predictors of unfavorable wound healing outcomes (Fig 5, Table 7), and the adjusted ORs were 0.058 (95% CI 0.004~0.938, P = 0.045) and 1.903 (95% CI 1.314~2.757, P<0.001), respectively.

**Table 6:** The relationship between higher NPS, lower PNI, and PWH.

		PWH		*χ* ^2^	*P*
		No	Yes		
PNI	high	60	1	13.556	**<0.001** *
	low	48	15		
Total		108	16		
NPS	low	83	4	17.897	**<0.001** *
	high	25	12		
Total		108	16		

* means statistical significance

**Table 7:** Stepwise logistic regression of the significant factors in the Univariate analysis.

	B	OR	95% CI	*P*
**Neutrophil count**	0.64	1.897	1.276~2.820	**0.002** *
**Serum albumin**	-1.128	0.324	0.147~0.711	**0.005** *
**Instrumented segments**	0.714	2.042	1.263~3.300	**0.004** *
**PNI**	-2.84	0.058	0.004~0.938	**0.045** *
**NPS**	0.644	1.903	1.314~2.757	**0.001** *

* means statistical significance

## Discussion

In the present study, we analyzed the relationship of the PNI and NPS with surgical incision healing prognosis at 1 month after thoracolumbar tuberculosis debridement and internal fixation. Through the univariate analysis and the stepwise logistic regression analysis it was revealed that the PNI and NPS might be independent risk factors for PWH. Meanwhile, the neutrophil count, the level of serum albumin, and the number of instrumented segments showed significant differences after all of the statistical analysis.

Tuberculosis (TB) is caused by Mycobacterium tuberculosis and is a major disease that seriously affects global public health. It is one of the world’s oldest diseases and one of the world’s top 10 causes of death, ranking first among infectious disease deaths, far ahead of the thrilling AIDS [28, 29]. Osteoarticular tuberculosis accounts for 1%-3% of all TB cases, and spinal tuberculosis is the most common form of osteoarticular tuberculosis, accounting for about 50% [30]. Tuberculosis of the spine (STB), also known as Pott’s disease, was first described in modern times by Percivall Pott in 1771 [31]. Spinal tuberculosis mainly occurs in the thoracolumbar segment, and tuberculosis often involves the vertebral body and intervertebral space, it is uncommon that spinal tuberculosis simply destroying the adnexa [7]. STB has the following characteristics: insidious onset, slow progression of the disease, non-specific clinical symptoms of patients, and low positive detection rate of TB bacillus culture test, so the missed diagnosis or misdiagnosis rate of spinal tuberculosis is high [32]. In addition, spinal tuberculosis patients are usually accompanied by malnutrition and poor immunity, and anti-tuberculosis drugs chemotherapy is not standardized. These factors result in increased difficulty in treatment [33–35].

In spite of 82–95% of patients with spinal TB can attain impressive clinical outcomes by anti-tuberculosis chemotherapy, there is still a risk of progressive neurological impairment [9]. In particular, thoracic TB has a great rate of disability and mortality, and surgical intervention is one of the critical methods to treat spinal TB [36]. Plus, the surgical treatment of spinal TB has been proven to be effective and beneficial to the prognosis of patients with spinal tuberculosis [37]. If the patient has: intractable pain or poor quality of life; Tuberculosis lesions increased, and progression was out of control; Spinal cord nerve compression appeared sensory and motor disorders; Deformity or segmental instability, should be timely surgical treatment [1]. The surgical procedure can be classified as radical or non-radical. Patients undergoing vertebral curettage with or without decompression or bone fusion with instrumentation could be called radical surgery. And those undergoing diagnostic operations, abscess drainage or curettage, debridement, anterior decompression, laminectomy, or discectomy should be defined as non-radical. But no distinction was made for surgical procedure, and we included both types in our study.

There is some evidence that malnutrition is associated with wound healing problems after an array of orthopedic surgeries [38, 39]. STB is a chronic wasting disease, and 25.7% of patients have pulmonary tuberculosis complications. These patients have high nutritional requirements, and the inflammatory and immune response caused by tuberculosis infection increases body consumption, but nutritional intake is not paid attention to make up, so preoperative malnutrition is very common in STB patients [40, 41]. Therefore, it is very important to regulate preoperative nutritional status of patients with spinal tuberculosis. The current nutritional assessment methods are divided into subjective and objective. The subjective assessment items are based on recent changes in nutritional intake, daily living ability and various symptoms of patients, such as the NRS-2002 score and the Subjective Global assessment (PG-SGA). Previous studies have shown that these assessment scales are significantly correlated with the prognosis of tuberculosis and can be used as prognostic indicators for tuberculosis patients [42]. Objective assessment, including preoperative laboratory test results and physical examination data, can reduce bias compared with subjective assessment, and can precisely and objectively evaluate preoperative nutrition. Prognostic nutritional index (PNI) can evaluate the nutritional status of patients undergoing surgery, predict the risk of surgery and evaluate the prognosis. It was established based on serum albumin and peripheral blood lymphocyte counts by Japanese scholars Onodera et al [43]. PNI was initially used to evaluate the nutritional and immune status of patients undergoing gastrointestinal surgery. In recent years, PNI has gradually become a new indicator to indicate the prognosis of patients with gastrointestinal malignancies, gynecological tumors and lung cancer. In addition, the prognostic prediction of non-tumor patients such as fracture, heart failure and cerebral infarction has also been applied more and more. Naples prognostic score (NPS), calculated from serum albumin, cholesterol, NLR and LMR, was initially carried out in the research of colorectal cancer by Galizia et al [23], recently has been widely researched in patients with malignancies, such as colorectal cancer, pancreatic cancer, endometrial cancer, lung cancer, gastric cancer, and esophageal squamous cell carcinoma. But PNI and NPS were rarely used in patients with spinal tuberculosis, especially in the evaluation of their postoperative incisional wound healing. In our study, although NPS has a larger area under the curve than PNI, both of these scoring systems could accurately predict postoperative wound healing before procedure. And high or low groups sectionalized via the cut-off value analyzed by ROC, high PNI group and low NPS group show a high synchronization (Table 5).

The projects that NPS and PNI share a common focus are albumin and lymphocytes. Serum albumin is a plasma protein produced by the liver, broadly indicated as albumins, including milk albumin, urinary proteins, and the secretion of the snail. Serum albumin is the most abundant protein in plasma, is a monomeric multi-domain macromolecule, representing the main determinant of plasma osmotic pressure and the main modulator of fluid distribution between body compartments [44]. It is the most commonly used and reliable evaluation marker of the nutritional status of the body [45]. It is well known that the incisional wound healing process involves the proliferation of injured fibroblasts and collagen synthesis, and albumin contributes to the development of this process. Bohl’s study found that patients with hypoalbuminemia had a higher risk of wound dehiscence, SSI, and urinary tract infection within the NSQIP database [46]. At the same time, our results indicated that albumin was an independent predictor for PWH. The AUC of albumin is 0.776 (95% CI 0.688–0.863, P < 0.001), and the threshold value is 39.5(g/L). That means when albumin is less than 39.5 (g/L), patients are more possible to develop PWH after surgery. The issue of preoperative malnutrition in STB patients was mentioned above. And one essential step of the procedure, debridement, is a fatal factor leading to lower the albumin level. Debridement also results in longer surgery time, more bleeding, and more trauma. Due to the physiological stress and inflammatory response after surgery, which increases capillary permeability, albumin in the blood vessels penetrates into the tissue space and reduces serum albumin, also be defined as transcapillary escape of albumin, hypoalbuminemia induce tissue edema and interstitial fluid leakage into the wound [47]. This can compromise the integrity of the wound, which closure. And intact innate and adaptive immune responses rely on albumin, PWH also provide a medium for bacteria to multiply, which unfortunately results in an increased risk of surgical site infection. Therefore, timely exogenous albumin supplementation is particularly important. Albumin supplementation could increase colloid osmotic pressure, increase plasma levels of antioxidants, and reduce inflammation [48]. Hence our findings recommend prompt interventions when a patient’s pre-operative albumin is below 39.5(g/L) to prevent postoperative unfavorable outcomes of wound healing.

The neutrophil count came out as an independent risk-predictive factor as well. Neutrophils are the first responders to trauma and infection. They target microbes and prevent their spread by producing active oxidants, activating particle components and neutrophils extracellular traps, pathways that are critical after trauma and infection and during incision healing [49]. The incision healing process is characterized by an essential inflammatory process at the beginning, in which a large number of neutrophils infiltrate the injured site [50]. Neutrophil extracellular traps (NETs), DNA-protein structures released by neutrophils, function as a defense mechanism, and their primary function is to trap and kill pathogens to defend against pathogen invasion in wounds [51]. However, it has been proved that it is harmful to the interaction of various cells and cytokines in the incision healing process, and its structure of it will directly damage the tissues around the incision and disrupt the highly coordinated healing process [52–54]. Thus, although NETs play an important role in pathogen defense, they can cause a delay in wound healing. Uncontrolled inflammatory response and excessive neutrophils lead to more NETs release, which may also be a risk factor for PWH in patients with STB after surgery. In this study, the AUC of neutrophil count is 0.789 (95% CI 0.648–0.930, P < 0.001), and the threshold value is 5.29 (10^9/L). Therefore, the neutrophils should be controlled within a reasonable range. That is, the inflammatory response should be regulated. We suggested a neutrophil count no more than 5.29 (10^9/L), which is conducive to postoperative incision healing.

Preoperative surgical plan based on the evaluation of the patient’s imaging data, symptoms, and signs. Incision of the corresponding skin and fascia was performed during the operation according to the plan. This study showed that the PWH rate increased significantly when the number of fixed segments was greater than 5 (AUC 0.756, 95% CI 0.605–0.907, P = 0.001). A larger number of surgically instrumented segments means a more considerable length of surgical incision, which causes more trauma to the body and increases the difficulty of healing. The wound healing process includes four processes: hemostasis, inflammation, proliferation, and tissue remodeling. It is a complex and dynamic process involving different cells and tissues [55]. Longer incisions require more fibroblast proliferation and collagen synthesis, and more supplementation of albumin. A systematic review by Noel et al. found that mean laparoscopic incision sizes were compared very favorably with open ones across 1,471 reviewed patients (6.9 vs. 17.6 cm) [56]. Wang et al. also showed in their study on the healing of Achilles tendon rupture surgical incisions that the recovery of shorter minimally invasive incisions (5 ~ 7 cm) generally got better prognosis than that of traditional incisions (14 ~ 15 cm) [57]. Shorten the length of incision to reduce the damage to the patient during the surgical operation is the standard minimally invasive concept. When compared to the typical degenerative disease of the spine, the procedure of thoracolumbar tuberculosis, of course, has such problems as long operation time, extensive surgical trauma, and more estimated blood loss. Therefore, patients with spinal tuberculosis should pay more attention to surgical trauma, and reduce the blow of surgery itself on patients. And we should bring new surgical techniques to this part through continuous practice and innovation in the future.

We also found a statistically significant difference in patients with pulmonary tuberculosis (PT) by univariate analysis in Fig 1 and Table 2, where patients with combined PT were more likely to develop PWH postoperatively. Patients with spinal tuberculosis are susceptible to complications of PT, and an epidemiologic study of STB patients based on demographic characteristics showed that 25.7% of 284 STB patients had complications of PT [41]. PT is associated with malnutrition and patients with PT are more likely to have hypoalbuminemia, with previous studies showing that 24% of patients with PT have concurrent hypoalbuminemia [58, 59]. Many studies have confirmed that serum albumin level is an independent and predictive risk factor for surgical site infection in spinal surgery [60, 61]. In our opinion, PWH in patients with spinal tuberculosis due to pulmonary tuberculosis may be caused by malnutrition of serum albumin in patients. Not only serum albumin level but also hemoglobin, plasma retinol and plasma zinc concentrations were lower in PT patients than in healthy individuals. [62] Overall malnutrition may be another crucial factor in the detrimental impact of PT on poor wound healing in STB patients. We should pay more attention to the nutritional status of STB patients with comorbid PT.

There are still some limitations in our research. We only assessed the preoperative nutritional status, and missed the postoperative nutritional status. This study was a retrospective single-center designed with a relatively small sample of patients. Our further studies, including prospective studies with more patients, are needed to assess the relationship between PNI and NPS and postoperative surgical incisional wound outcomes in patients with STB. And explore more potentially meaningful predictors at the same time. And the study was conducted on a specific group of patients in a particular hospital, mainly for consistency in terms of the surgeon and surgical technique. But are these results from patients in our hospitals generalizable to other patients around the world needs to be confirmed to the best of our ability in future studies.

## Conclusion

To our knowledge, this is the first study to evaluate the wound prognostic role of preoperative NPS and PNI in STB patients. In conclusion, higher NPS and lower PNI might be associated with a poor wound outcome in patients with STB. The NPS and PNI, which are inexpensive and readily available biomarkers, may be helpful in identifying patients with poor prognoses who will benefit from early nutritional therapy.
